# Effect of TGF-β1 transfected dental pulp derived mesenchymal stem cells on *ın vivo* cartilage regeneration

**DOI:** 10.22038/ijbms.2025.87715.18946

**Published:** 2025

**Authors:** Betul Tekin Alpargu, Saim Ozdamar, Zeynep Burcin Gonen, Ayca Kara, Hasan Salkin

**Affiliations:** 1 Department of Histology and Embryology, Faculty of Medicine, Erciyes University, Kayseri, Türkiye; 2 Department of Histology and Embryology, Faculty of Medicine, Pamukkale University, Denizli, Türkiye; 3 Department of Oral and Maxillofacial Surgery, Genome and Stem Cell Center (GENKOK), Erciyes University, Kayseri, Türkiye; 4 Genome and Stem Cell Center (GENKOK), Erciyes University, Kayseri, Türkiye; 5 Department of Histology and Embryology, Faculty of Medicine, Istanbul Beykent University, Istanbul, Türkiye

**Keywords:** Cartilage regeneration, Dental pulp, Mesenchymal stem cells, Osteochondral defects TGF-β1

## Abstract

**Objective(s):**

This study aimed to investigate the effect of TGF-β1-transfected dental pulp-derived mesenchymal stem cells (DP-MSCs) on the regeneration of osteochondral defects in rabbit knee joints.

**Materials and Methods:**

A total of 32 New Zealand white rabbits (3-4 months old, 2-3 kg) were randomly divided into four groups (n=8 per group). Osteochondral defects were surgically created in the distal femoral articular cartilage of three experimental groups, while the control group remained untreated. Group 2 received an intra-articular injection of 0.5 ml sterile saline. Group 3 was administered 0.5 ml of saline containing 3×10^6^ DP-MSCs. Group 4 received 3×10^6^ TGF-β1-transfected DP-MSCs suspended in 0.5 ml of saline. After six weeks, animals were euthanized, and femoral joints were harvested. Tissue sections (5 μm) were analyzed histologically using hematoxylin-eosin, Masson’s trichrome, and Toluidine blue staining, as well as immunohistochemical methods.

**Results:**

Histological analysis revealed increased fibrous cartilage in the DP-MSC group compared to the saline group, with some irregular hyaline cartilage regions and dispersed chondrocytes. The TGF-β1+DP-MSC group demonstrated a more significant improvement, characterized by enhanced hyaline cartilage formation and a more organized tissue architecture.

**Conclusion:**

TGF-β1 transfection enhances the chondrogenic potential of mesenchymal stem cells by stimulating reparative cellular mechanisms and promoting the formation of hyaline cartilage, thereby facilitating more effective tissue regeneration. These findings suggest that this strategy holds considerable potential for clinical application in cartilage repair and regeneration.

## Introduction

Articular cartilage (AC) covers the diarthrodial joints and plays a key role in the mechanical distribution of loads across the joints. The structure and function of the AC are primarily regulated by chondrocytes, which control the transformation of the extracellular matrix (ECM) and maintain tissue homeostasis. Disruption of chondrocyte function can result in degenerative diseases, such as osteoarthritis (OA). Cartilage degeneration is a result of chondrocyte hypertrophy, accompanied by the expression of proteolytic enzymes (1-3). While chronic AC damage typically develops during the progression of OA, acute cartilage or osteochondral injuries can arise from a single traumatic event or a displaced intra-articular fracture.

The connection between OA and AC damage is significant, making the clinical advancement of cartilage regeneration highly important. Unlike spontaneous OA, which generally affects middle-aged and older individuals, post-traumatic OA (PTOA) caused by cartilage injury often impacts younger individuals. The optimal treatment for these cases is to regenerate the damaged AC in order to restore the original joint function, instead of resorting to joint replacement or arthrodesis. There is an increasing focus on less invasive treatment options for both acute and chronic AC lesions, as these approaches can effectively address the problem without requiring more invasive procedures (4). Today, various methods are used for cartilage repair, with cellular treatment approaches gaining significant attention in recent years. Stem cells derived from various sources have been shown to be effective in treatment in different studies (5, 6). However, due to the limited cartilage regeneration, there are ongoing debates about the effectiveness of using MSCs in treatment. As a result, it was suggested that activating MSCs might lead to faster and more effective treatment, which prompted the initiation of studies on the transfection of different genes into MSCs.

The process of chondrogenesis is influenced by a variety of hormones, growth factors, cytokines, and transcription factors (7). The TGF-β superfamily, insulin-like growth factor, fibroblast growth factor, platelet-derived growth factors, and bone morphogenetic proteins play a crucial role in the chondrogenesis of MSCs (8). TGF-β1 is a key growth factor for chondrogenic differentiation of mesenchymal stem cells (9). It was observed that cell activity increased after gene transfection into MSCs, resulting in their differentiation in a chondrogenic direction. TGFB1-treated MSCs promote ECM remodeling, differentiation, and molecular responses, which enhance their adhesive properties (10). Additionally, SOX9 is a crucial transcription factor for chondrogenesis, ensuring chondrocyte lineage commitment, promoting cell survival, and activating genes essential for the production of cartilage-specific components. It also regulates the expression of key matrix proteins, including type II, IX, and XI collagen, as well as aggrecan, which are crucial for maintaining cartilage structure and function (11, 12).

Recent studies have identified various adult stem cell populations in diverse tissues, including skin, cartilage, intestine, blood, dental pulp, and mammary epithelial cells. Among these, human dental tissues, including dental pulp, dental follicle, periodontal ligament, and bone marrow, have emerged as promising sources of mesenchymal stem cells (MSCs). Under suitable conditions, dental tissue MSCs have the potential to differentiate into multiple cell types, including neural cells, osteocytes, and chondrocytes (13). Additionally, the differentiation of dental pulp stem cells (DPSCs) is regulated by various regulators that control osteogenesis, such as the TGF-β superfamily and various cytokines. Therefore, mesenchymal stem cells (MSCs) derived from the oral cavity represent an invaluable resource for the development of future clinical-grade cells (14).

In conclusion, dental pulp stem cells offer several advantages, including a rich stem cell population and ease of collection, which eliminates the need for additional surgical procedures, making them a promising alternative to bone marrow and other tissue-derived stem cells. However, numerous studies have demonstrated that mesenchymal stem cells are an effective source for osteogenic differentiation. In contrast, studies investigating the chondrogenic differentiation of mesenchymal stem cells from dental pulp have been limited (10). Therefore, this study aims to assess the impact of TGF-β1-transfected dental pulp mesenchymal stem cells (DP-MSCs) on the regeneration of knee AC defects in rabbits. For this purpose, we conducted a histological evaluation of cartilage damage. We compared the expression patterns of SOX9, COL, and AGG across the different experimental groups, control, damaged, DP-MSCs, and TGF-β1-transfected DP-MSCs. The primary aim of this experimental study is to demonstrate the impact of TGF-β1-transfected dental pulp mesenchymal stem cells (DP-MSCs) on the regeneration of knee AC defects in rabbits, comparing the effects with those observed in rabbits treated with only DP-MSCs.

## Materials and Methods

### DP-MSCs culture and characterization and TGF-β1 transfection

In a study previously conducted by Salkin *et al*. (15), rabbit dental pulp stem cells were isolated from the upper and lower incisors using collagenase and dispase. Cells stored in a liquid nitrogen tank were extracted and thawed in a 37 ^°^C water bath, having been characterized at the second passage. Cells were cultured in alpha MEM with nucleosides, supplemented with 20% FBS, and incubated at 37 ^°^C in a 5% CO₂ atmosphere. 

After Passage 3 (P3), the rabbit DPSCs were removed from the flasks by trypsinization and were washed twice with Ca^2+^ and Mg^2+^ containing PBS (Biological Industries, #02-020-1A, Israel Beit Haemek). To study the antibodies of CD29-FITC Conjugated (EMD Millipore Corp., #FCMAB269F, USA), primer antibody CD44 (Bio-Rad Lab., Inc., #MCA2504, USA) and primer antibody CD45 (Bio- Rad Lab., Inc., #MCA808GA, USA), cells were gathered in PBS containing Ca^2+^ and Mg^2+^ (Biological Industries, #02- 020-1A, Israel Beit Haemek) at 1x10^6^ cells in three tubes. The cells were fixed with 4% paraformaldehyde (Merck #104005, USA) and incubated at room temperature for 10 min. Antibodies were added to the cells as the CD29-FITC Conjugated antibodies (EMD Millipore Corp., #FCMAB269F, USA), CD44 primer antibodies (Bio-Rad Lab., Inc., #MCA2504, USA), and CD45 primer antibodies (Bio-Rad Lab., Inc., #MCA808GA, USA) with 1:100 dilution, and were incubated at room temperature. The cells treated with CD29, CD44 (Bio-Rad Lab., Inc., #MCA2504, USA), and CD45 (Bio-Rad Lab., Inc., #MCA808GA, USA) primer antibodies were centrifuged after incubation, and Goat anti-Mouse secondary antibody (Abcam, #ab7064, UK) was added. The cells were then incubated with the FITC-labeled secondary antibody in the dark for 30 min. Flow cytometry was performed using FACS Canto II (BD Biosciences, San Diego, USA) (15). 

Once the cells reached 70% confluency, they were harvested by trypsinization and expanded for the DP-MSCs and TGF-β1+DP-MSCs groups. TGF-β1-transfected cells were obtained pre-prepared from a previous study (15), conducted at the Genome and Stem Cell Center. The cells were cultured in alpha-modified Eagle’s medium containing 20% fetal bovine serum (FBS). The TGF-β1 plasmid was introduced into these cells via electroporation, and cells that stably integrated the gene were selected using selective antibiotics. These genetically modified cells were then cultured and applied to animals according to the experimental groups.

### Evaluation of transfection efficiency

To demonstrate the transfection efficiency, TGF-β1 protein expression was compared in cells transfected with a TGF-β1 plasmid and untransfected cells. To compare TGF-β1 protein expression in cells, cells were immunofluorescently stained with a primary antibody against TGF-β1 and β-actin. Then, TGF-β1 protein expression in transfected and non-transfected cells was compared using a confocal microscope (Zeiss, Germany).

### Animals

The study involved 32 male New Zealand rabbits, aged 3 to 4 months and weighing 2-3 kg, which were obtained from a certified experimental and clinical research center. All animals were provided with care following standard ethical guidelines. The Institutional Animal Research Ethics Committee granted ethical approval for the study. All procedures were conducted under the TYL-2016-6991 project, in compliance with the relevant Institutional Experimental Animal Ethics Committee, dated August 24, 2016, and numbered 16/107.

### Surgical protocol

**Figure 1:**
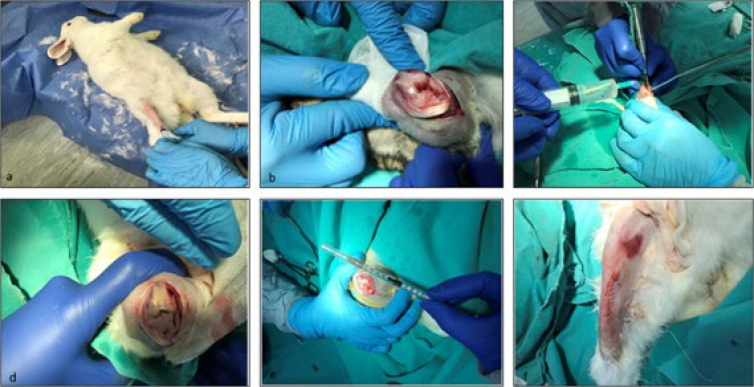
Representation of experimental design. Created by BioRender

**Figure 2:**
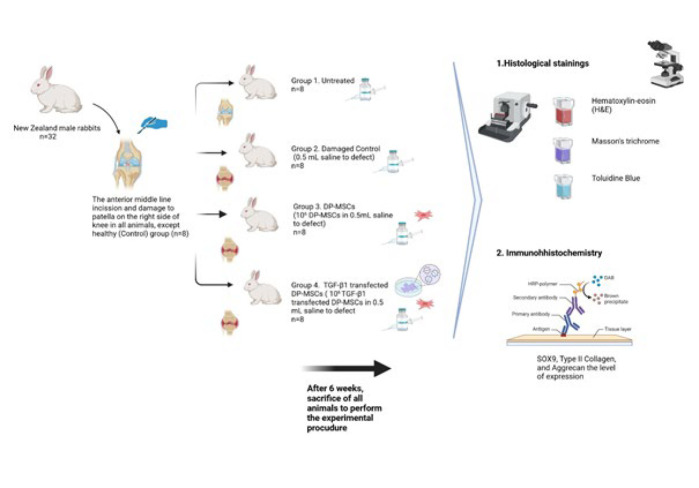
Presentation of the animal defect model and demonstration of dental pulp mesenchymal stem cells (DP-MSCs) applications in tissue engineering, regenerative therapy, and cartilage repair

General anesthesia was induced with 35 mg/kg of ketamine (Pfizer, 022114, USA) and 5 mg/kg of Rompun (Bayer, 81082405, Turkey), administered via intramuscular injection. After skin preparation and disinfection, an anterior midline incision was made to expose the patella in the right knee of all the animals. The patella was laterally dislocated to expose the femoral articular surface. Standard osteochondral defects, measuring 5 mm in width and 6 mm in length, were created on the weight-bearing surface of the distal medial femoral condyle using a hand drill bit (Figure 2). A total of 32 rabbits were distributed among different experimental groups. The experimental design is already shown in Figure 1.

After the surrounding soft tissue was carefully treated, the surgical wound was closed in layers to promote proper healing. A single dose of intramuscular antibiotics (Cefazolin, 25 mg/kg) (Pfizer, RDC10752152, USA) and analgesic (Carprofen, 4 mg/kg) (Santa Cruz, sc-395914Rx, USA) was given to prevent infection and manage pain. The wound was disinfected with beta-iodine once daily (Figure 2).

### Histological analysis

All animals were sacrificed for the extraction of the femurs at the end of six weeks. Joint cartilage tissue from sacrificed rabbits was fixed with a formaldehyde solution (Merck, 104002 Germany) of 4% concentration. Tissues waiting for 48 hr in the fixation solution were then left in the decalcifying agent, nitric acid solution (Sigma, 438073, USA), for 1 week to 10 days, as controlled. At the end of six weeks, all animals were sacrificed for the extraction of the femurs. The joint cartilage tissue from the sacrificed rabbits was fixed in a 4% formaldehyde solution (Merck, 104002, Germany). After being stored in the fixation solution for 48 hr, the tissues were then immersed in a decalcifying agent, nitric acid solution (Sigma, 438073, USA), for 1 to 10 days, with careful oversight. The nitric acid solution was prepared by mixing the components (distilled water, nitric acid, and formalin) in a fixed ratio of 8:1:1, respectively. After decalcification, the tissues were left overnight in running tap water for further processing. The tissues were then passed through an increasing series of alcohol solutions, cleared with xylol, and embedded in paraffin and blocked. Sections of 4-5 μm were cut from the paraffin blocks and placed on polylysine-coated slides. The paraffin from the prepared sections was removed using standard histological techniques, including treatment with xylol (Merck, 108684, Germany), and the sections were then passed through a graded series of alcohol solutions. The sections were examined by staining with hematoxylin-eosin (H&E) (Hematoxylin; Merck 109253, Germany, Eosin; Carlo Erba 446632, Spain), Masson’s trichrome stain (Fuchsin acid; Merck, C.I. 42685, Germany, Phosphomolybdic acid; Sigma, 221856, Germany, Aniline blue; Sigma, 28631-66-5, Germany), and Toluidine Blue (Merck 89640, Germany) to observe the general histological structure.

### Wakitani’s cartilage repair scoring system

Histological sections of the tissue were analyzed to evaluate cartilage repair in the experimental groups. A grading system with five modified categories—cell morphology, matrix staining, surface regularity, cartilage regularity, and integration into the surrounding cartilage—was used to assess the recovery of cartilage damage, as defined by Wakitani and colleagues (Table 1)(12). The control group was assigned a score of 0 (zero), and the recovery status of the other groups was observed. If their values were closer to 0, the improvement was considered greater. Scoring was conducted by two observers, with no significant difference between their assessments. SOX9, Col-II, and Agg Gene expression levels between groups were evaluated by using the ImageJ program. Additionally, the lengths of the damage area and the recovery area were measured using the ImageJ program.

### Immunohistochemistry

In our study, the avidin-biotin-peroxidase method was used to assess the expression of SOX9, Type II Collagen, and aggrecan in the AC tissue obtained from rabbits. The 5-μm-thick sections taken from paraffin blocks on poly-L-lysine-coated slides were dried overnight in an oven at 60 ^°^C. They were then rehydrated by passing through xylene and decreasing concentrations of alcohol (100%, 96%, 80%, 70%). The sections were then washed three times in distilled water for 5 minutes each. For antigen retrieval, the sections were placed in a container on an electric hob, ensuring heat transmission, and the temperature was set to 82 ^°^C for approximately 30 min. To cool the slides, the container was transferred to another container filled with cold water, and the water was changed twice to facilitate effective heat exchange. The preparations, after being washed with distilled water for 5 minutes, were then rinsed with phosphate-buffered saline (PBS)(Sigma, P4417, USA). The sections were treated with 3% hydrogen peroxide for 20 min to block endogenous peroxidase activity, followed by three washes with PBS, each for 5 minutes. In the subsequent stages, the Large Volume Detection System (Thermo Scientific, TP-125-HL, USA) immunohistochemistry staining kit was used. To minimize background staining, the sections were treated with Ultra V block for 5 minutes, followed by overnight incubation with primary antibodies (SOX9 (Abcam, ab185966, USA), COL-II (Abcam, ab34712, USA), AGG (Abcam, ab3773, USA)) at 4 ^°^C in a humid environment. For the negative control tissues, PBS was used instead of the primary antibody, and the following steps were performed in the same manner. Cartilage tissue was also used as a positive control. After incubation with the primary antibody, the sections were rinsed. Reverse staining was performed using Gill’s Hematoxylin (Sigma, GHS132, USA) after the biotinylated secondary antibody, Streptavidin-HRP, and DAB (3,3’-Diaminobenzidine)(Thermo Scientific, TA-125-HDX, USA) chromogen. Finally, the sections were dehydrated by immersion in a graded series of alcohol (70%, 80%, 96%, 100%), passed through xylene, and covered with Entellan® (Merck, 107961, Germany), a rapid mounting medium, before being observed under an Olympus BX51 microscope.

### Statistical analysis

For statistical analysis, the normal distribution of the data was evaluated using histograms, Q-Q plots, and the Shapiro-Wilk test. The Kruskal-Wallis test was applied to compare quantitative variables among more than two groups. Spearman correlation analysis was used to evaluate the relationship between quantitative data. For multiple comparisons, the Dunn-Bonferroni test was employed. Data were analyzed using the Turcosa Cloud (Turcosa Ltd. Co.) statistical software. The levels of significance are indicated as follows: *P*˂0.05 *, P˂0.01 **, P˂0.001 ***.

## Results

The animals did not experience any infections at the surgical wound sites. Furthermore, there was no separation or reopening of the wound edges. This suggests that the healing process occurred without complications such as infection or wound disruption, indicating a smooth recovery.

### DP-MSCs characterization and TGF-β1 transfection efficiency

**Figure 3:**
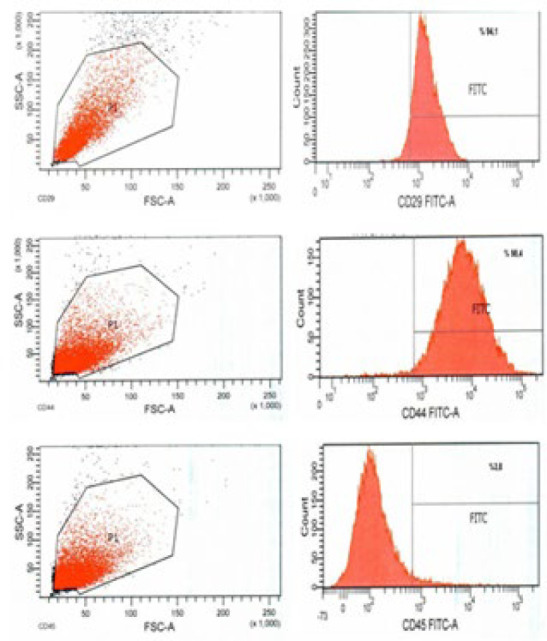
Flow cytometry findings showed that rabbit DP-MSCs positively expressed CD29 in 94% and CD44 in 99%

**Figure 4:**
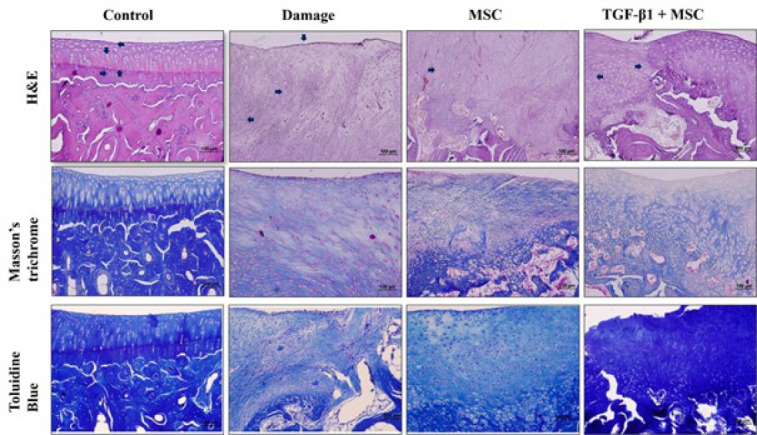
Our confocal microscope findings showed that TGF-β1 expression was overexpressed in TGF-β1-transfected rabbit DP-MSCs compared to non-transfected DP-MSCs

Flow cytometry analysis revealed that the isolated rabbit dental pulp stem cells were positive for CD29 and CD44, and negative for CD45. Thus, these cells were immunophenotypically shown to be mesenchymal stem cells (Figure 3). Our confocal microscope findings showed that TGF-β1 protein expression was significantly increased and overexpressed in rabbit DP-MSCs after TGF-β1 transfection compared to non-transfected cells. These findings demonstrated that the TGF-β1 plasmid was successfully transferred into rabbit DP-MSCs (Figure 4).

### Light microscopic findings

**Figure 5:**
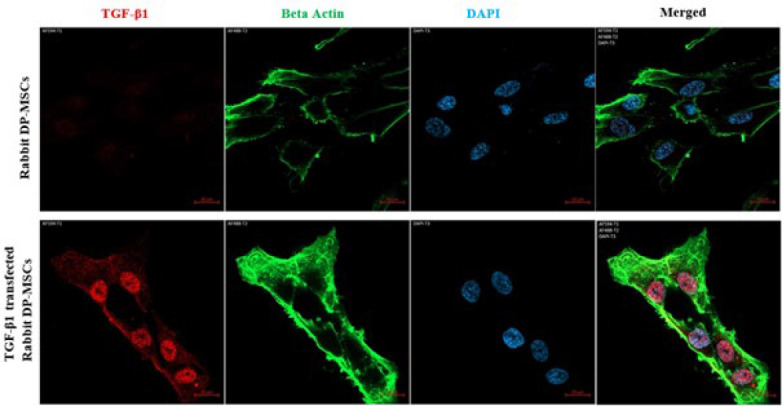
Light microscopy of cartilage repair in experimental groups (H&E, Masson’s trichrome, Toluidine blue)

In our study, the repair of rabbits with osteochondral defects (A width of 5 mm and a length of 6 mm) was evaluated by a light microscope. The differences between the groups were assessed based on the damage group, and the evaluation was carried out using five categories. The tissues from the groups were examined using hematoxylin-eosin, Masson’s trichrome, and toluidine blue stains. As a result of the histomorphological evaluation, the AC in the control group had a smooth appearance. The chondrocytes in the superficial zone appeared as discs between the collagen layers, were more spherical in the transition zone, and were vertically aligned in the deep zone along the joint line. The boundary between the deep zone and the calcified zone was observed as a straight line, known as the Tidemark Line (Figure 5). In the SF-treated group, which was the damaged group, small areas of self-healing were observed in some regions, with connective tissue primarily consisting of irregular collagen fibers (Figure 5). There was an increase in the amount of fibrous cartilage and a decrease in the amount of connective tissue in the DP-MSCs group compared to the damage group. Additionally, some areas showed an irregular hyaline cartilage structure with scattered chondrocytes, forming distinct regions (Figure 5). Pannus structure was observed in almost all samples of the damage group. While this structure remained in some samples of the DP-MSCs group, it was absent in some samples of the TGF-β1+DP-MSCs group. However, an increase in the amount of hyaline cartilage and fibrous cartilage was still observed in the TGF-β1+DP-MSCs group compared to the DP-MSCs group. Due to the high cellular density, it is believed that production is still ongoing. Although the chondrocytes were densely clustered and disorganized, there were occasionally organized areas (Figure 5). There was a significant difference in the TGF-β1+DP-MSCs group in terms of cellular density, cartilage regularity, integration into the surrounding tissue, and matrix staining when compared to the other groups (*P*˂0.05 *, Table 2).

According to the results of the statistical analysis, a significant difference was observed in cartilage regularity and integration into the surrounding cartilage tissue. Upon further evaluation, it was found that the TGF-β1+DP-MSCs group showed significantly higher results compared to the damage group. However, there was no significant difference between the damage group and the DP-MSCs group, nor between the DP-MSCs group and the TGF-β1+DP-MSCs group. In the assessment, it was assumed that healing would be more effective if the scores were closer to zero (0). To evaluate the degree of healing in the damaged area, sections from the damage group, DP-MSCs group, and TGF-β1+DP-MSCs group were analyzed at (x4) magnification. The length of the damaged or healed area was quantified using the Image J program, and the results of the statistical analysis are presented in Table 3. When the length measurements of the damaged or healing areas were analyzed, a highly significant difference was observed in the TGF-β1+DP-MSCs group, with greater values in the area, maximum value, and length compared to the Damage group. However, no significant differences were found between the Damage group and the DP-MSCs group, or between the DP-MSCs group and the TGF-β1+DP-MSCs group.

### Immunohistochemical findings

**Figure 6:**
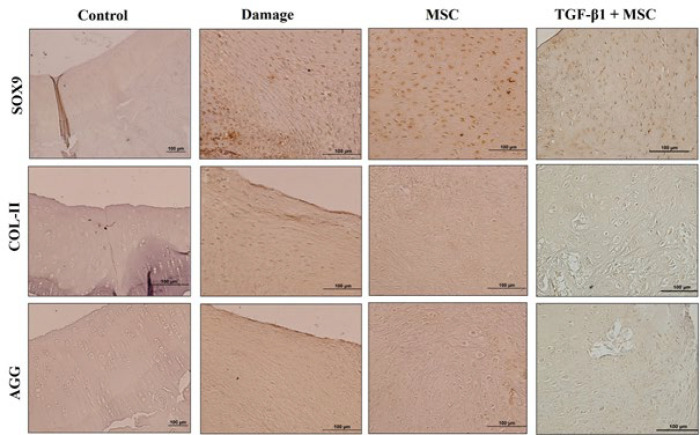
Representative images showing the expression of SOX9, COL-II, and AGG, arranged from top to bottom, respectively

Immunohistochemical staining was performed to evaluate the gene expression of SOX9, a crucial transcription factor in embryonic development, along with COL-II and AGG, essential extracellular matrix (ECM) components that contribute to chondrocyte formation and the development of the cartilage matrix. These analyses were conducted on tissue sections from the damage group, DP-MSCs group, and TGF-β1+DP-MSCs group. The mean intensity levels of the tissue sections were quantified, revealing no expression in the negative control tissues of each group. SOX9 expression was evaluated in the chondrocyte cells, COL-II expression was assessed within the connective tissue areas, and AGG expression was examined in both the lacustrine and connective tissue regions of the chondrocytes. The expression levels of SOX9, COL-II, and AGG were compared to those observed in the negative control, as shown in Figure 6. The results of the statistical analysis are presented in Table 4.

A significant difference was observed across all gene expressions. Specifically, SOX9, COL-II, and AGG gene expressions in the TGF-β1+DP-MSCs group were significantly higher compared to both the damage group and the DP-MSCs group. No significant difference was observed between the damage group and the DP-MSCs group.

### Associating with nonparametric correlation between groups and variables

The statistical analysis revealed several significant relationships between variables in the damage group. A strong positive correlation was found between the expressions of SOX9 and COL-II genes (rho=0.743, *P*=0.035). A robust negative correlation was observed between AGG expression and peripheral cartilage tissue integration (rho=-0.802, *P*=0.030). Additionally, a moderate positive relationship was identified between the mean value of cell morphology and the damage field length measurement (rho=0.768, *P*=0.044). A significant positive relationship was also found between matrix staining and cartilage regularity (rho=0.764, *P*=0.046). A robust negative correlation was noted between cartilage regularity and the angle values in the damage area length measurement (rho=-0.802, *P*=0.030). Lastly, a moderate negative relationship was observed between peripheral cartilage tissue integration and the minimum values of the damage area length measurement (rho=-0.757, *P*=0.049). These findings suggest meaningful associations between the various variables in the context of tissue damage.

In addition to the previous findings, the statistical analysis revealed several significant relationships in the DP-MSCs group. A robust positive correlation was observed between matrix staining and surface regularity (rho=0.913, *P*=0.030). Similarly, a powerful positive relationship was found between surface regularity and integration into peripheral cartilaginous tissue (rho=0.884, *P*=0.047). Furthermore, a robust positive correlation was noted between the maximum values of the healing area length and integration into peripheral cartilaginous tissue (rho=0.894, *P*=0.041). These results indicate meaningful associations between matrix staining, surface regularity, and tissue integration in the healing process within the DP-MSCs group.

Finally, the statistical analysis revealed several significant relationships in the TGF-β1+DP-MSCs group. A moderate negative correlation was found between SOX9 and AGG gene expression (rho=-0.714, *P*=0.047). Additionally, a moderate positive relationship was observed between SOX9 and the healing area measurement (rho=0.755, *P*=0.031). A moderate negative correlation was also noted between SOX9 and the angle value of the healing measurement (rho=-0.714, *P*=0.047). Furthermore, a moderate positive relationship was found between SOX9 and the length value of the healing measurement. These results highlight meaningful associations between SOX9 expression and various aspects of tissue healing within the TGF-β1+DP-MSCs group.

## Discussion

The reconstruction of the extracellular matrix (ECM) is a delicate balance in which old proteins are continuously degraded and new ones are synthesized, a process crucial for maintaining healthy tissue function (16). This balance can be disrupted in connective tissue diseases, leading to alterations in tissue structure, often characterized by a shift in the processes of formation and degradation. The irreversible destruction of the cartilage collagen network plays a key role in the pathophysiological progression of arthritis. During tissue remodeling, proteases generate small protein fragments that can serve as serological biomarkers for tissue degradation (17).

In a study by Kouri et al. (18), OA fibrils and non-fibrillar tissues exhibited cell clustering effects. Cells in the damaged area showed increased proliferation and were characterized by clustering. Additionally, the study highlighted the presence of abundant phyllopodia, primary cilia, and changes in the cytoskeleton. These findings suggest the active movement of chondrocytes toward the damaged areas. Moreover, a recent study indicates that chondrocytes or chondroprogenitor cells migrate to the injury site, where they repair the damage by synthesizing the lost ECM. To facilitate this movement, cells degrade the surrounding ECM through the expression of proteolytic enzymes and amoeboid locomotion (19).

In our study, we aimed to observe cellular density in the damage area by creating a localized injury in the group treated solely with SF. We observed clustering of connective tissue cells and the formation of chondrocytes in our damaged group. However, in a related study, the differentiation and aggregation of chondroprogenitor cells from the synovial mesenchymal stem cell niche for cartilage repair were described (20, 21). Synovial cells were induced to differentiate into chondrocytes when cultured on BMP-coated plates (22), highlighting the influence of growth factors such as TGF-β / BMP on synovial cells. These factors may lead to the differentiation and migration of synovial stem cells to AC as an attempt to repair damaged cartilage tissue in OA (23). In general, our study demonstrated a greater cellular density in the group treated with TGF-β1-transfected DP-MSCs compared to the other groups, as observed in the histomorphological analysis. 

Typically, foundational cartilage tissue engineering studies are conducted in vitro to better understand cellular behavior and tissue regeneration processes. The transition to in vivo animal models is influenced by factors such as the suitability of the model and associated costs. Small animal models, including mice, rats, and rabbits, have been commonly used in early studies. Rats and rabbits, in particular, have sufficiently large joints that allow for surgical manipulation, making them popular choices for cartilage repair studies. In fact, the cartilage of these animals is thinner than that of humans, and their biomechanical properties and knee joint load rates may differ from those of humans, with the potential for spontaneous recovery of cartilage damage. While thousands of studies have been published on human cartilage over the past 25 years, there are only 11 studies involving bovine animals, including sheep and pigs. Small animal models, such as rabbits and rats, are generally preferred in research due to their practicality and ease of use in experimental settings (24). Although rabbits are considered a practical model for the initial stages of assessment due to their cost-effectiveness, ease of use, and reasonably sized joints, a notable difference from humans is the intrinsic healing ability of rabbit cartilage, which is often observed as a feature not present in human cartilage (25). In our study, conducted with a rabbit model, we observed intrinsic healing centers in the damage group that received SF, similar to the observations made in previous studies. Furthermore, the rabbit models used in our study allow for the monitoring of cellular changes after injection and provide a longer follow-up period, enabling the collection of more detailed information about the fate of the cells, their contributions, and the induction of OA.

The field of translational medicine in the medical world is an emerging area of research. A key issue in this field is whether human cells can be effectively transferred to patients, or if cells derived from animals are safe for patient use. This raises important questions about the safety, efficacy, and ethical considerations surrounding cell-based therapies. In light of these discussions, we used cells derived from rabbit tooth pulp (allogenic) as a stem cell source in our study. This approach aimed to explore the potential of using animal-derived cells for therapeutic purposes, while considering the safety and efficacy of such treatments in the context of translational medicine. 

MSCs are the preferred cell type for articular cartilage tissue engineering due to their ease of isolation, expansion, and growth. These cells possess multi-lineage differentiation potential, particularly for chondrogenic differentiation, making them an ideal candidate for cellular therapy in the treatment of articular cartilage damage (26). Moreover, the immunomodulatory and anti-inflammatory properties of MSCs have demonstrated their potential as an excellent candidate for cell therapy in the treatment of inflammatory diseases such as OA and AR (27).

Current treatment methods for OA offer limited success, primarily providing temporary relief from pain. Surgical procedures, including debridement, drilling, osteochondral transplantation, and autologous chondrocyte implantation, also achieve only limited success. Research into articular cartilage is a relatively recent endeavor, and there remains much to be understood about the normal development of the synovial joint, its components, and their interactions in the context of osteoarthritis and focal cartilage defects (28), Karlsson et al. (29) demonstrated the presence of a progenitor cell population in the Ranvier perichondral groove as well as in the joint cartilage of the knee, exhibiting characteristics of a stem cell niche. However, this progenitor cell population is insufficient to induce spontaneous regeneration in superficial lesions of the articular cartilage. Grogan et al. (30), in their recent studies, have shown that a larger number of cells in human OA cartilage express progenitor cell markers compared to normal cartilage. However, the percentage of stem cells in OA cartilage was found to be lower than the expected density. Due to the increasing interest in the clinical application of MSCs for treatment, their use in the treatment of OA in knee joints has become more widespread in recent years. Determining the optimal number of MSCs required for effective treatment of OA, along with investigating the potential use of additional triggers such as hyaluronan, remains an active area of research. Lamo-Espinosa et al. (31) demonstrated that in vitro autologous BM-MSCs were a safe and feasible procedure, leading to clinical and functional recovery in knee OA following a single intra-articular injection of HA. These results pave the way for future clinical trials. In our study, building upon previous research, we considered the phase III status of clinical trials involving the administration of DP-MSCs and hypothesized that gene therapy could be a valuable addition to clinical studies. Therefore, we aimed to highlight the growing importance and potential of gene therapy methods in the context of cartilage repair and osteoarthritis treatment.

The TGF-β superfamily plays a critical role in various biological processes and effective mechanisms within the cell. Key functions include the differentiation of chondrocytes and regulation of their de-differentiation, maintenance of cartilage homeostasis, and the regulation of cartilage synthesis. It also influences collagen and proteoglycan synthesis, enhances extracellular matrix biosynthesis, and supports local cartilage repair, fracture healing, and renewal of OA lesions. Additionally, TGF-β contributes to angiogenesis, vascularization, lower jaw development, and the regulation of communication between different cell types. It also plays a role in the phenotypic transformation of fibroblasts, controls physical processes during wound and bone healing, and regulates steroid synthesis (32). Today, TGF-β3 is widely used as a standard for chondrogenic differentiation of stem cells. In a study by Rizk et al. (33), it was shown that transfecting the TGF-β3 gene into human dental pulp stem cells successfully induced chondrogenic differentiation. However, in a study by Salkin et al. (15), TGF-β1 was also identified as a potent inducer of chondrogenesis in stem cells. While TGF-β1 is more commonly associated with bone differentiation, it is also known to play a significant role in early chondrogenesis before bone formation, as it induces the differentiation of cartilage in the early stages of skeletal development and during bone formation. Liu, Ping et al. (34) demonstrated that chondrogenic differentiation occurred in rabbit BM-MSCs when cultured in a TGF-β1-supplemented medium. Furthermore, TGF-β1 was found to facilitate the growth and maturation of chondrocytes, while also enhancing the aggregation of type II collagen and proteoglycans, which are essential components of the extracellular matrix in cartilage.

In this study, the gene expression levels of SOX9 and AGG were evaluated over time, with results showing that both gene expression levels varied according to the days (15). In our study, a negative, strong, and significant correlation was found between the gene expression of SOX9 and AGG in the relationship between variables and groups. Therefore, we believe that the observed difference in expression between SOX9 and AGG may be attributed to the different stages of chondrogenesis and matrix formation. This suggests that further studies should explore this difference in more detail. SOX9 is a key transcription factor that regulates chondrogenesis; however, its role in the chondrogenic differentiation of mesenchymal stem cells induced by various carriers or factors remains poorly understood. Additionally, one study suggested that collagen could mediate SOX9 expression by providing a biomimetic microenvironment that supports cell concentration prior to chondrogenesis (11). SOX9 is irrevocable for the expression of chondrogenic cell markers and chondrocyte-specific matrix proteins, including type II, IX, and XI collagen, as well as aggrecan. Furthermore, the expression of SOX9 is significantly reduced in individuals with osteoarthritis, particularly in age-related cartilage degeneration, when compared to controls (35). Cartilage-specific matrix proteins are crucial for maintaining the biomechanical properties of articular cartilage. Static compressive forces have been shown to trigger the expression of type II collagen and aggrecan, along with an increase in SOX9 expression (36). These findings indicate that SOX9 has potential clinical value. The essential role of SOX9 in cartilage formation and its involvement in osteochondroprogenitor loss, chondrogenic mesenchymal condensation, and the proper proliferation, differentiation, maturation, and hypertrophic transformation of chondrocytes were demonstrated using mutant mice. Additionally, the SOX9-SOX5 and SOX6 form was identified as the primary regulatory mechanism of chondrogenesis. Overexpression of SOX9, SOX5, and SOX6 in cultured cells (37), as well as ectopic expression of SOX9 in mice, has been shown to induce type II collagen expression (38, 39). This evidence clearly demonstrates that the SOX9-SOX5 and SOX6 are the main regulator of chondrogenesis. In our study, when examining the correlation between variables and groups, as well as the relationship between variables in the damage group, we found a positive, strong, and significant relationship between SOX9 and COL-II gene expression. Therefore, we believe that there is a supportive mechanism between the SOX9 gene and COL-II, indicating a potential regulatory interaction in cartilage repair. In our study, histomorphological analysis showed that the damage group primarily exhibited a cellular density composed of connective tissue cells, and while some improvement was observed in the damage area, complete recovery was not achieved. In the DP-MSCs group, there was an increase in cellular distribution compared to the damage group, though this increase was not statistically significant. However, in the TGF-β1 transfected DP-MSCs group, the addition of the TGF-β1 growth factor promoted cartilage formation, suggesting a potential enhancement in cartilage repair. We have demonstrated that TGF-β1 increases cellular density by triggering chondrocyte formation, enhances the integration of cartilage with surrounding tissue, and plays an active role in the healing process, helping to minimize the damaged area. Additionally, immunohistochemical staining results revealed no significant difference between the damage group and the DP-MSCs group, but a significant difference was observed between the damage group and the TGF-β1 + DP-MSCs group. The correlation analysis of these results highlighted the importance of expression time periods. Based on these findings, we anticipate that further studies can build upon and improve these results.

Group 1: Untreated group (n=8), Group 2: Damaged control group (n=8), 0.5 ml saline was applied to each defect, Group 3: DP-MSCs group (n=8), 106 DP-MSCs were applied in 0.5 ml saline to each defect, Group 4: TGF-β1-transfected DP-MSCs group (n=8). 10^6^ TGF-β1-transfected DP-MSCs were applied in 0.5 ml saline to each defect

The images sequentially illustrate the following stage: (a) preparation of the joint by shaving and administering anesthesia for the experiment, (b) visualization of the surface of the healthy joint, (c) induction of damage to the joint surface, (d) observation of cartilage tissue damage, (e) transfer of stem cells to the tissue through the synovial area, (f) completion of the process with surgical stitching after procedure

**Table:** 

Categories	Features	Skoring
1-Cell morphology	Hyaline cartilage Mostly hyaline cartilage Mostly fibrocartilage Mostly non-cartilage Non-cartilage	0 1 2 3 4
2-Matriks staining intensity	Normal Slightly reduced Markedly reduced No metachromatic stain	0 1 2 3
3-Surface regularity	Smooth Moderate Irregular Severely irregular	0 1 2 3
4- Thickness of cartilage	Smooth Moderate Irregular Severely irregular	0 1 2 3
5-Integration of donor with host cartilage	Both edges integrated One edge integrated Neither edges integrated	0 1 2

CD45 was negative in rabbit DP-MSCs

Control group, X20, H+E, Masson trichrome, Toluidine blue. The first row of light microscopic images represents a robust cartilage tissue that includes different types of chondrocytes, such as chondroblasts (the right side arrow), maturing chondrocytes, and mature chondrocytes (the left side arrow). Additionally, line of chondrocytes in single file, Tidemark line (the upside arrow), the isogen groups (the down side arrow) formed by cells. Damage group, X20, H+E, Masson trichrome, Toluidine blue. The second row light microscobic images represent damage group forming the connective tissue consisting of dense collagen with the fields of the fibrous (the right side arrow), pannus structures (the down side arrow), which prevents the development of the AC on the surface of finish line, in cartilage and intrinsic healing area, fibrous cartilage (the left side arrow). MSC group, X20, H+E, Masson trichrome, Toluidine blue. The third row light microscopic images represent the hyaline cartilage area (right side arrow), pannus structures (down side arrow) that prevent the development of AC, the area of mature chondrocytes, and the isogenic group of cells. TGF-β1+MSC group, X20, H+E, Masson trichrome blue, Toluidine blue. The fourth row light microscopic images represent integration with donor host cartilage tissue (the right side arrow), the matrix density of hyaline cartilage tissue (the left side arrow), and a myriad of mature and maturing chondrocytes. Scale bar: 100 μm

**Table 2:** Statistical analysis of histomorphological scoring results

Histomorphological measurements	Groups			*P*
	The Damage Group (n=7)	The MCS Group (n=5)	The TGF-β1+MCS Group (n=8)	
	(25 p / 75 p)	(25 p / 75 p)	(25 p / 75 p)	*P*>0.05
Cell morphology	2,0 ( 2,0 / 3,0)	1,0 ( 1,0 /2,0 )	1.0 ( 1,0 / 2,0 )	
Matrix Staining	2,0 ( 2,0 / 2,0)	2.0 ( 1,0 / 2,0)	2.0 ( 1,0 / 2,0 )	*P*>0.05
Surface regularity	3,0 ( 2,0 / 3,0)	2.0 ( 1,0 / 3,0)	2,0 ( 2,0 / 3,0)	*P*>0.05
Thickness of cartilage	3,0 ( 2,0 / 3,0)^a^	2,0 ( 2,0 / 3,0)^a,b^	2,0 ( 1,0 / 2,0)^b^	*P*˂0.05*
Integration of donor with host cartilage	2,0 ( 1,0 / 2,0)^a^	0,0 ( 0,0 / 2,0)^a,b^	0,0 ( 0,0 / 1,0)^b^	*P*˂0.05 *

Data are presented as median (Q1-Q3 percentile). Comparisons were made among the Damage Group (n=7), the MCS Group (n=5), and the TGF-β1+MCS Group (n=8). No significant differences were observed between groups for cell morphology, matrix staining, or surface regularity (*P>*0.05). However, significant differences were detected in cartilage thickness and donor–host cartilage integration (*P<*0.05*). Superscript letters (a, b, ab) indicate statistical differences between groups, while the same letters in the same row represent the similarity between the groups. The levels of significance are indicated as follows: *P˂*0.05 *, *P˂*0.01 **, *P˂*0.001 ***

**Table 3:** Comparative statistical analysis results of damage area and recovery area length across different treatment groups

Damage recovery area measurement	Groups			*P-value*
	Damage Group (n=7)	MCS Group (n=5)	GFβ1+MKH Group (n=8)	
	(25 p / 75 p)	(25 p / 75p)	(25 p / 75 p)	
Area	1324,0 (985,0 /1671,0) ^a^	883,0 (643,0 /1055,0) ^a, b^	796,0 (681,3 /883,3) ^b^	*P*˂0.05 *
Mean	148089,0 (123319,0 /160258,0)	149037,0 (131387,0 (177294,0)	143044,0 (81579,3 /163479,0)	*P*>0.05
Min	63879,0 (49957,0 /76530,0)	73083,0 (38085,0 /117140,0)	93881,0 (41894,0 /140825,3)	*P*>0.05
Maks	194836,0 (187427,0 /203421,0) ^a^	197616,0 (186923,0/208063,0) ^a, b^	176528,0 (130239,0 /186888,0) ^b^	*P*˂0.05 *
Angle	-90339,0 (-91052,0 /-89039,0)	-90339,0 (-91052,0 /-89039,0)	-89973,0 (-90768,3 /-88705,0)	*P*>0.05
IntDent	20152589,0 (138335305,0/242650837,3)	111421657,0 (91417562,0/186512903,0)	113738830,0 (58423417,3/137228300,0)	*P*>0.05
Length	1323064,0 (984270,0 /1669771,0)^a^	882326,0 (642028,0 /1053672,0) ^a, b^	795056,0 (680060,0/882203,0) ^b^	*P*˂0.05 *

Data are presented as median (Q1-Q3 percentile). Statistical comparisons were performed using the Kruskal–Wallis test followed by the Dunn–Bonferroni *post hoc* test for multiple comparisons. Differences were considered statistically significant at *P<*0.05. Significant reductions in damage area, maximum values, and recovery length were observed in the MCS and TGFβ1+MKH groups compared to the Damage Group (*P<*0.05*). No significant differences were found in mean, minimum, angle, or IntDent measurements (*P>*0.05). Superscript letters (a, b, ab) indicate statistically significant differences between groups; the same letters in a row represent groups that are not significantly different. The levels of significance are indicated as follows: *P<*0.05 *, *P<*0.01**, *P<*0.001 ***

SOX9 expression was localized to chondrocytes, COL-II was predominantly found in the connective tissue, and AGG was observed both around chondrocyte lacunae and within the connective tissue. Negative control samples showed no detectable expression in any group. Statistical analysis revealed significant differences in the expression of SOX9, COL-II, and AGG among the groups (*P<*0.05). The TGF-β1+MSC group exhibited significantly increased expression compared to the Damaged and MSC groups, while no significant difference was observed between the Damaged and MSC groups. Scale bar=100 μm

**Table 4:** Statistical analysis results of gene expression levels (SOX9, AGG, COL-II) across treatment groups

Gene Expression measurements	Gruplar			*P*
	Damage group (n=8)	MKH group (n=8)	TGF-β1+MKH group (n=8)	
	Ort (25 p / 75 p)	Ort (25 p / 75 p)	Ort (25 p / 75 p)	*P*˂0.01 **
SOX9	0,0 ( 0,0 / 0,1) ^a^	0,0 ( 0,0 / 0,1) ^a^	0,2 ( 0,2 / 0,2) ^b^	
AGG	0,1 ( 0,1 / 0,1) ^a^	0,1 ( 0,1 / 0,1) ^a^	0,3 ( 0,3 / 0,3) ^b^	*P*˂0.01 **
COL-II	0,1 ( 0,1 / 0,2) ^a^	0,1 ( 0,0 / 0,1) ^a^	0,3 ( 0,3 / 0,3) ^b^	*P*˂0.01 **

Gene expression levels are presented as median values with interquartile ranges (Q1-Q3 percentile). Comparisons were made between the Damage group (n=8), MKH group (n=8), and TGF-β1+MKH group (n=8). The TGF-β1+MKH group demonstrated significantly higher expression of SOX9, AGG, and COL-II compared to both the Damage and MKH groups (*P<*0.01**), which did not differ significantly from each other. Statistical significance was determined using appropriate non-parametric tests, with significance thresholds indicated as *P<*0.05*, *P<*0.01**, and *P<*0.001***. Superscript letters (a, b, ab) indicate statistical differences between groups, while the same letters in the same row represent the similarity between the groups

## Conclusion

This study highlights the dynamic expression of SOX9 and AGG during chondrogenesis, revealing a strong negative correlation between these genes, likely reflecting distinct stages of cartilage matrix formation. Our findings suggest that SOX9, a pivotal transcription factor regulating chondrogenesis, plays an essential role in mesenchymal stem cell differentiation and cartilage repair. The observed positive correlation between SOX9 and COL-II gene expression further supports a potential regulatory interaction crucial for cartilage regeneration. 

Histomorphological analysis demonstrated limited recovery in the damage group, with increased cellularity observed following DP-MSC treatment, though this was not statistically significant. Notably, transfection with TGF-β1 significantly enhanced cartilage formation, increased chondrocyte density, and promoted integration with surrounding tissue, thereby facilitating repair and reducing damaged areas. Immunohistochemical results corroborated these findings, showing significant differences in gene expression only in the TGF-β1+DP-MSC group compared to controls. 

Collectively, these results underscore the importance of SOX9-mediated pathways and TGF-β1 in cartilage repair. Our data suggest a potential regulatory mechanism between SOX9 and COL-II during cartilage regeneration. The timing of gene expression emerges as a critical factor influencing repair outcomes. Based on these findings, further research is warranted to elucidate the underlying mechanisms and to optimize therapeutic strategies for cartilage regeneration.

## Declaration

AI tools were used solely for proofreading purposes to improve the language and clarity of the manuscript. No AI-generated content was used in the generation of scientific data or results.
